# Spatiotemporal proteolytic susceptibility of allergens: positive or negative effects on the allergic sensitization?

**DOI:** 10.3389/falgy.2024.1426816

**Published:** 2024-07-09

**Authors:** Alain Jacquet, Wai Tuck Soh

**Affiliations:** ^1^ Department of Biochemistry, Faculty of Medicine, Chulalongkorn University, Bangkok, Thailand; ^2^ Research Group of Quantitative and Systems Biology, Max-Planck-Institute for Multidisciplinary Sciences, Göttingen, Germany; ^3^ Faculty of Applied Sciences, UCSI University, Kuala Lumpur, Malaysia

**Keywords:** allergen, protease, antigen processing, proteolysis, lipid-binding activity

## Abstract

From their expression in their respective allergenic source to their processing by antigen presenting cells, allergens continuously encounter proteases. The ability of allergens to resist to proteolysis by digestive enzymes or host-cell/microbial proteases is considered as an important property that influences their allergenic potential. However, the relationship between proteolytic stability and allergenicity is much more complex and depends on various factors, such as the protein structure dynamics, the exposure level, the route of sensitization, and their respective protease susceptibility. In this review, we summarize and discuss the current knowledge on several aspects of allergen proteolytic stability in different environments including the allergenic sources, routes of sensitization (skin, respiratory tract, gastrointestinal tract) and endolysosomal compartment of antigen-presenting cells. Proteolytic stability alone cannot represent a definitive criterion to allergenicity. The proteolytic susceptibility of allergens in processed extracts can affect allergy diagnosis and immunotherapy. Furthermore, the fine tuning of allergen stability during antigen processing can be exploited for the development of novel immunotherapeutic strategies.

## Introduction

The key question “what makes an allergen an allergen?” remains, up to now, partially answered, rendering the distinction between allergenic and non-allergenic proteins difficult. It is reasonable to think that allergen abundance and stability are important factors favoring the initiation of the allergic sensitization (1). However, the relationship between these two properties and allergenicity is far from straightforward. As allergens are proteins, they are susceptible to meet proteolytic enzymes during their lifetime, from their expression to their processing, mainly by dendritic cells, for their presentation to T cells. Consequently, when exposed to endogen or environmental proteases from the skin, airways, gastrointestinal tract, a certain level of proteolytic stability of allergens is at least required to ensure their survival in order to maintain their antigenic properties. Several factors can affect the proteolytic susceptibility of allergens including environmental pH, structure dynamics, the presence of bound ligands and post-translational modifications (2–5). In addition, the proteolytic stability of allergens is also relevant to the diagnosis and treatment of allergy. Indeed, the presence of proteases in the extracts alters the allergen composition over time, affecting the accuracy of skin prick tests, specific IgE assays, oral food challenges, and immunotherapy. Here, we review and discuss several aspects of proteolytic stability of allergens, in their natural environment, during the skin, airway, gastrointestinal exposures and finally, in the course of their endolysosomal processing by antigen-presenting cells. The different effects of proteases on some specific allergens are summarized in Table 1.

**Table 1:** Impacts of proteolytic susceptibility of allergens.

Allergen	Proteolysis effect	Outcome/potential impact	References
Natural Bet v 1 in pollen extracts	Bet v 1 fragmentation	Impact on IgE assay for the diagnosis of allergic sensitization	(6)
Bla g 1/Bla g 2, Amb a 1, Der p 1/Der p 2 in respective allergen extracts	Degradation upon storage at room temperature or 37 °C	Impact on IgE assay for the diagnosis of allergic sensitization	(7–9)
Recombinant Der p 5 and 13	Proteolysis by trypsin	Absence of Der p 5 and Der p 13 in HDM allergen extracts following proteolysis by trypsin-like protease Der p 3	(10, 11)
Der p 23	Cleavage by Der p 1	Intact Der p 23 concentration in HDM allergen extracts affected	(12)
ProDer p 3, 6, and 9	Proteolytic maturation by Der p 1	Increase in serine protease activity in HDM allergen extracts	(13)
ProDer p 1	Autocatalytic maturation under acidic conditions	Allergenicity enhanced	(14, 15)
Der p 1 and 3	Autolysis	Allergenicity decreased Impact on IgE assay for the diagnosis of allergic sensitization	(14)
Amb a 11	Autocatalytic maturation under acidic conditions	Allergenicity enhanced	(16)
Hev b 6.01	Post-translational cleavages	Allergen fragmentation into N-terminal hevein (Hev b 6.02) and C-terminal Hev b 6.03	(17, 18)
Vicilin family (Jug r 2, Cor a 11, Pis v 3 or Ana o 1)	Maturation by asparaginyl endopeptidase	IgE reactivity enhanced	(19–21)
Act d 5	Post-translational cleavages by cysteine protease Act d 1	Allergen fragmentation into an N-terminal domain kissper and C-terminal domain KiTH	(22)
Ara h 2, 3 and 6	Post-translational cleavages by endogenous peanut proteases	Maturation of allergen Allergenicity enhanced?	(23, 24) (25)
Bos d 5	Degradation by pepsin	Allergenicity decreased	(26)
Bos d 4	Degradation by Pepsin	Allergenicity decreased	(27)
Bos d 4 and 5 in pasteurized and dried skim milk	Higher resistance to gastric enzymes	Allergenicity enhanced	(28)
Gal d 1 and 2	Digestion by pepsin	Allergenicity decreased	(29, 30)
Ara h 1 and 3	Digestion by pepsin	Allergenicity decreased	(31)
Shrimp tropomyosin	Digestable at pH <2.5, Resistant to pepsin at pH >4	Allergenicity increase Allergenicity enhanced	(32)
Pru p 3	Gastrointestinal proteolysis Bile salts amplify the proteolysis	Allergenicity decreased	(33)
Ara h 8 in roasted peanuts	Higher resistance to pepsin treatment	Allergenicity increased	(34)
Ara h 1 and Sin a 2	Higher gastric proteolytic resistance by binding phosphatidyl glycerol acid	Proteolytic stability increased by lipid-allergen interaction	(35)
Bos d 4 and 5	Higher gastroduodenal proteolytic resistance by binding phosphatidyl choline	Proteolytic stability increased by lipid-allergen interaction	(26, 27)
Fold stabilized Bet v 1 mutants	Proteolysis by lysosomal proteases reduced	Allergenicity increased	(3)
Holo forms of Bet v 1	Change in proteolytic resistance to lysosomal proteases, cathepsin S or legumain	Proteolytic stability dependent on lipid-allergen interaction	(4)
Nitrated Bet v 1	Unknown	MHC Bet v 1-derived peptide presentation increased	(36)
Oxidized Bet v 2	Resistance to cathepsin S degradation increased	Unknown	(37)
Fold stabilized Phl p 6 mutant	No change in endolysosomal protease susceptibility	Shift from Th2 to Th1/Th17 polarization	(5)

## Protease susceptibility of allergens in allergen extracts or recombinant allergen preparations

Since proteases are ubiquitous and have important physiological tasks in all living organisms (animals, plants, microorganisms), any allergenic source contains a large variety of proteolytic enzymes. Some of them are even classified as protease allergens which can have critical roles in the initiation and regulation of the allergic inflammation (38). The presence of allergenic and non-allergenic proteases in allergenic sources can deeply influence the allergen stability, affecting the longevity of allergens as intact and functional proteins (39, 40). For instance, a proteomic profiling of birch pollen extracts revealed the presence of truncated forms of Bet v 1 (6). Despite the presence of endogenous and microbial proteases in birch pollen extracts (40), no investigation so far has been done to measure the impact of these proteases on the allergenic composition of these extracts. While the temporal fate of the expressed allergens from any source remains poorly elucidated, it is reasonable to suggest that proteolytic activities of non-allergenic proteases or protease allergens could drastically affect the concentration as well as the composition of the extracts, leading to issues in standardization of extracts for the diagnosis and treatment by allergen immunotherapy. Several reports supported this hypothesis. Stability studies of allergen extracts showed extensive to complete deprivations of Bla g 1/Bla g 2, Amb a 1, Der p 1/Der p 2 (from cockroach, ragweed, house dust mite respectively) when extracts are stored at room temperature or 37 °C (7–9). The addition of protease inhibitors extends the shelf life of *P. americana* (cockroach) extract, demonstrating that the allergen degradation was protease dependent (7). It is worth noting that recombinant forms of HDM allergen Der p 5 and Der p 13 can be completely digested by trypsin within an hour (10, 11). Consequently, the trypsin-like activity of Der p 3, an abundant protease in HDM extracts (41), may potentially affect the concentration of Der p 5, Der p 13 or other susceptible mite allergens. In addition, recombinant Der p 23 was also shown to be truncated by Der p 1 but not trypsin, indicating a specific proteolytic event (12). We also observed that recombinant Der p 7 is cleaved by Der p 1, whereas recombinant Der p 21 is completely degraded by Trypsin (unpublished data).

## Protease effects on allergen maturation

One of the best examples showing the role of proteases in the maturation of allergens is the orchestration of Der p 3, Der p 6, Der p 9 (trypsin-, chymotrypsin-, and collagenolytic-like proteases respectively) serine protease processing by cysteine protease HDM allergen Der p 1 (13). Once the autocatalytic maturation of ProDer p 1 into active Der p 1 occurs under acidic conditions in the mite midgut, Der p 1 subsequently converts the zymogens ProDer p 3, ProDer p 6 and ProDer p 9 into active forms in the hindgut at pH 6. It must be pointed out that the removal of the prosequence increases the Der p 1 and Der p 3 allergenicity by exposing IgE binding epitopes (14, 15). However, contrary to Der p 1, Der p 3 can be degraded by autolysis (14). The cysteine protease allergen Amb a 11 from ragweed pollen can be autocatalytically matured *in vitro* at pH 5 (16). The rubber latex allergen Hev b 6.01 (prohevein), following post-translational cleavages, is converted into two allergenic fragments: the N-terminal hevein (Hev b 6.02) and the C-terminal Hev b 6.03 (17, 18). Vicilin is a family of cysteine-rich seed storage proteins found in many allergenic food sources such as peanuts, legumes, fruits, and grains. The vicilin allergens such as Jug r 2, Cor a 11, Pis v 3 or Ana o 1 are expressed with an N-terminal leader sequence, which contains a variable number of IgE reactive vicilin-buried peptides derived from the parent vicilin during the maturation process mediated by asparaginyl endopeptidase (19–21). The kiwi fruit allergen Act d 5 is a kiwellin that can be converted by the cysteine protease Act d 1 (actinidin) into two IgE binding domains: an N-terminal domain called kissper (a pore forming peptide) and a larger C-terminal domain called KiTH (22). The major peanut allergens Ara h 2 and Ara h 6 are members of the 2S albumin protein family. Both allergens undergo a proteolytic processing by endogenous peanut proteases leading to the C-terminal removal of a dipeptide for Ara h 2, the formation of N- and C-terminal subunits for Ara h 6 (23, 24). Full-length Ara h 3 protein is cleaved to create acidic and basic subunits linked by an intermolecular disulfide bridge (25).

## Food allergen stability and gastroduodenal digestion

Since food allergens reach the gastrointestinal tract during sensitization, we generally assume that these proteins must be highly abundant and resistant to proteolytic degradation in order to initiate the allergic response. Linear IgE binding epitopes are supposed to play an important role in food allergy as the probability is high for the loss of conformational epitopes during the food heating and/or the passage through the gastrointestinal tract. The food allergen digestion takes place first in the stomach by pepsin under highly acidic pH. These acidic conditions can also facilitate the unfolding of allergens. When the food bole enters the intestine, the proteolytic processing of food allergens is achieved by the activity of pancreatic secreted trypsin, chymotrypsin, carboxypeptidase A and B, enteropeptidases and elastase.

A large body of evidence has undermined the dogma of digestive stability (42, 43). The correlation between stability to proteolytic digestion and allergenicity has not been verified: food allergens and non-allergenic proteins do not necessarily differ in terms of proteolytic resistance. However, the assessment of allergenicity remains to be evaluated in *in vitro* gastric (and duodenal) digestion assays, which simulate gastrointestinal digestion (44). The results of these studies are largely dependent on the allergen/protease molar ratio (level of exposure), pH, incubation time (timing of exposure), and food processing, leading to potential misinterpretation. Moreover, food allergens are not consumed alone but are commonly associated with protein-food matrices which could influence the digestibility of the allergens, further affecting the assessment (45). Despite these issues, such assays continue to be used by official agencies such as the EFSA (European Food Safety Agency) for the safety assessment of new food proteins and notwithstanding the lack of correlation between *in vivo* human digestion of proteins and their allergenicity. The situation is even more complex, as epicutaneous exposure to food allergens has been shown to be critical for the initiation of allergic sensitisation (46). Gastric and duodenal digestion assays represent then only one piece of the puzzle to qualify food proteins as allergens.

The major milk allergen, Bos d 5 (β-lactoglobulin), is a dimeric lipocalin capable of transporting a wide range of lipid cargos and it is considered as the most resistant allergen to proteolysis in raw milk. Actually, whereas Bos d 5 remained stable following a 2 h treatment with pepsin, a 15 min simulated duodenal digestion triggers allergen degradation into peptides (26). Whey allergens Bos d 4 (α-lactalbumin) incubated with pepsin for 15 min is fully converted into <6 kDa peptides (27). The stability of milk allergens to gastric digestion was at least dependent on the milk processing methods. Bos d 4 and Bos d 5 display a reduced allergenicity with heat treatment at temperatures above 85 °C whereas caseins are thermostable. Bos d 4 and Bos d 5 in pasteurized and dried skim milk showed a higher resistance to gastric enzymes compared to the same proteins found in ultra-heat-treated milk (28). For egg allergens, pepsin-digested ovalbumin (OVA; Gal d 2) and ovomucoid (OVM; Gal d 1) reportedly had lower IgE-binding activities than the intact proteins (29, 30). According to the size of the identified peptides (Maximal Mw: <1.5 kD, 15 amino acids), these data could suggest that ingested Gal d 1 and Gal d 2 cannot trigger allergenic sensitization. For IgE-cross linking, to induce mast cell degranulation, the peptides need to be larger than 3 kDa or a minimum length of 30 amino acids (47). Peanut allergens Ara h 2 and Ara h 6, two 2S albumin, are quite resistant to gastric digestion (48). Digestion with pepsin and endocytosis-mediated transport through intestinal epithelium reduced the capacity of Ara h 1 and 3, but not of Ara h 2 and 6 to activate mast cells (31). The digestibility of shrimp tropomyosin is highly pH-dependent: Tropomyosin can be digested at pH lower than 2.5 whereas this allergen is resistant to pepsin at pH values higher than 4 (32).

## Influence of lipid binding on allergen stability

It is well established that the allergenicity of proteins displaying lipid/fatty acid binding capacity results from the adjuvanticity properties of their lipid cargos (49). Furthermore, these lipid ligands can reduce the proteolytic sensitivity of these categories of allergens. This is particularly evident for Bla g 1 (major cockroach allergen) and Bet v 1 (major birch pollen allergen), where binding of lipids affected their conformation dynamics, resulting in differential proteolytic susceptibility to lysosomal proteases (2, 4). We will further discuss the effect of stability during lysosomal processing in the next section of this review.

Non-specific Lipid Transport proteins (nsLTPs) are pan-allergens largely present in plant foods. These allergens which can bind multiple lipid ligands are particularly resistant to gastric digestion (50). This suggested that sufficient amount of intact LTPs could be transported through the intestinal epithelium to initiate the allergic sensitization (51). Grape or sunflower LTP showed higher resistance to gastric digestion when the assay was performed in the presence of phosphatidyl choline (52). Upon linoleic acid lipid binding, the susceptibility of Pru p 3 (peach LTP) to duodenal digestion remained unchanged whereas the wheat LTP digestibility was increased (53). Of note, the gastrointestinal proteolysis of Pru p 3 was increased in the presence of bile salts (33), suggesting that the nature of the lipid ligands influences the allergen stability to proteases.

The peanut allergen Ara h 8, a member of Bet v 1-like family, was shown to be more resistant to pepsin treatment when peanuts were roasted (34). This food processing might induce a lipid-allergen complex improving the allergen stability. We can also speculate that the association of Soy Allergens (cysteine protease Gly m Bd 30K, 7S Globulin Gly m 5, 11 S globulin Gly m 6) with oil bodies in the plant might influence their proteolytic susceptibility (54). The binding of phosphatidyl glycerol acid into the hydrophobic pocket of peanut Ara h 1 (7S Globulin) and mustard Sin a 2 (11S globulin) conferred gastric protease resistance (35). Addition of phosphatidyl choline greatly increased Bos d 4 and Bos d 5 resistance to gastric and gastroduodenal conditions respectively (26, 27).

## Proteolytic susceptibility of allergens during antigen processing

Antigen processing and presentation pathways by antigen-presenting cells (mainly dendritic cells) are essential for the initiation and regulation of adaptive immune responses. They involve the uptake and degradation of antigens, and display of the resulting antigenic peptides by major histocompatibility complex (MHC) molecules to T cells. The antigen processing and presentation pathways were extensively reviewed by Pishesha, et al. (55). As allergens are exogenous antigens, the captured allergens are compartmentalized into endolysosomes which gradually undergo acidification. This pH change initiates the allergen processing by lysosomal proteases, e.g., cathepsin S, B, L, and legumain (56). The resulting peptides can then be loaded onto MHC-II and present to CD4^+^ T-helper cells (55).

Endolysosomal protease susceptibility of allergens drastically influences epitope presentation and the subsequent T-cell responses. Studies have shown that an increase in protein stability or slow proteolytic degradation can have either enhancement (57–59) or detrimental (60–62) effect on antigen processing and presentation, thereby affecting the allergenicity of allergens. In birch pollen, multiple isoforms of the major allergen Bet v 1 were identified and their sequence identity was higher than 95%. The most abundant isoform Bet v 1.0101 (Bet v 1a) is considered as hyperallergenic because birch pollen allergic patients are predominantly sensitized to this variant (63). Bet v 1.0101 was shown to be more resistant to cathepsin S digestion than Bet v 1.0102 (Bet v 1d), an isoform displaying reduced IgE reactivity (64). The difference in protease susceptibility is explained by a higher exposure of Bet v 1.0102 amide backbone to solvent, leading to a more extended degradation by cathepsin S (64, 65). However, it is unclear how the difference in degradability by lysosomal proteases can affect the derivation of Bet v 1-specific peptides available for MHC-II presentation. The importance of structural fold stability in the Bet v 1a immunogenicity was further investigated using several Bet v 1a mutants designed from *in silico* prediction and characterized experimentally through thermal stability assay and structural determination by x-ray crystallography. The fold stabilized mutants had a reduction in proteolytic degradation by lysosomal proteases *in vitro* and were more immunogenic in mice (by inducing higher level of IgG1, IgE and IL-4 secreting splenocytes) as compared with wildtype Bet v 1a (3).

Bet v 1 displays a hydrophobic cavity that can harbor various type of pollen-derived hydrophobic cargos such as flavonoids, phytohormones, and microbe-derived compounds (4, 66). The presence of different ligand in Bet v 1 resulted in different degree of proteolytic resistance to lysosomal proteases, cathepsin S or legumain (4). Most of the ligands have a stabilizing effect to Bet v 1. In addition, mass spectrometry analysis of the aforementioned digestion assays also revealed a remarkably change in the dynamic of peptides repertoire generation. Taken together, these data evidenced that the binding of ligands influences Bet v 1 structural stability, thereby changing the susceptibility to proteases. Surprisingly, a pollen-derived Bet v 1 ligand, E_1_ phytoprostane, was found not only to enhance Bet v 1 proteolytic stability to lysosomal proteases but also exerted an inhibition effect to cathepsin S, leading to a reduction in the presentation of a known Bet v 1 T-cell epitope (4). Of note, the binding of lipid cargo to major cockroach allergen Bla g 1 similarly led to a higher resistance to cathepsin S, thus, resulted in poor T-cell epitope generation (2). Collectively, these studies revealed that extrinsic factors (ligand binding and/or biological activity of ligand) play a role in modulating allergen stability during antigen processing and potentially influence the allergenicity and immunogenicity of allergens.

Besides, post-translational modifications (PTMs) are contributing factors of the allergen proteolytic stability during antigen processing. The antigen processing of a chemically induced nitrated Bet v 1.0101 (a PTM mimicking the effect of nitrogen dioxide and ozone on Bet v 1 in polluted environments) was also found to increase the presentation of Bet v 1-derived peptides (36). Another birch pollen allergen, Bet v 2 (profilin), was found to exist in both oxidized (with an intra-disulfide bond) and reduced form (without disulfide bond). The oxidized form is more resistant to cathepsin S degradation albeit both forms share similar IgE reactivity and basophil activation capacity (37). However, whether the proteolytic resistance of oxidized Bet v 2 leads to higher MHC-II presentation remains to be investigated.

Structural rigidification through point mutations of the grass pollen allergen Phl p 6, resulted in a shift in immune polarization from Th2 to Th1/Th17 in mice. Importantly, stabilization/destabilization did not change the repertoire of presented peptides both *in vivo* and *in vitro*. However, the presentation of an immunodominant epitope from a destabilized Phl p 6 mutant was more efficient than the one from highly stable mutant or wild-type form (5).

Collectively, given that the allergen structural stability during proteolysis could tune T-cell polarization, these findings pave the way to design an interesting vaccine strategy to desensitize allergic patients. Indeed, engineered fold destabilized variants of Bet v 1 (BM4) (67, 68) and Der p 2 (S47W) (69) were found to promote immune tolerance in preclinical studies (70).

## Perspectives

**Figure 1:**
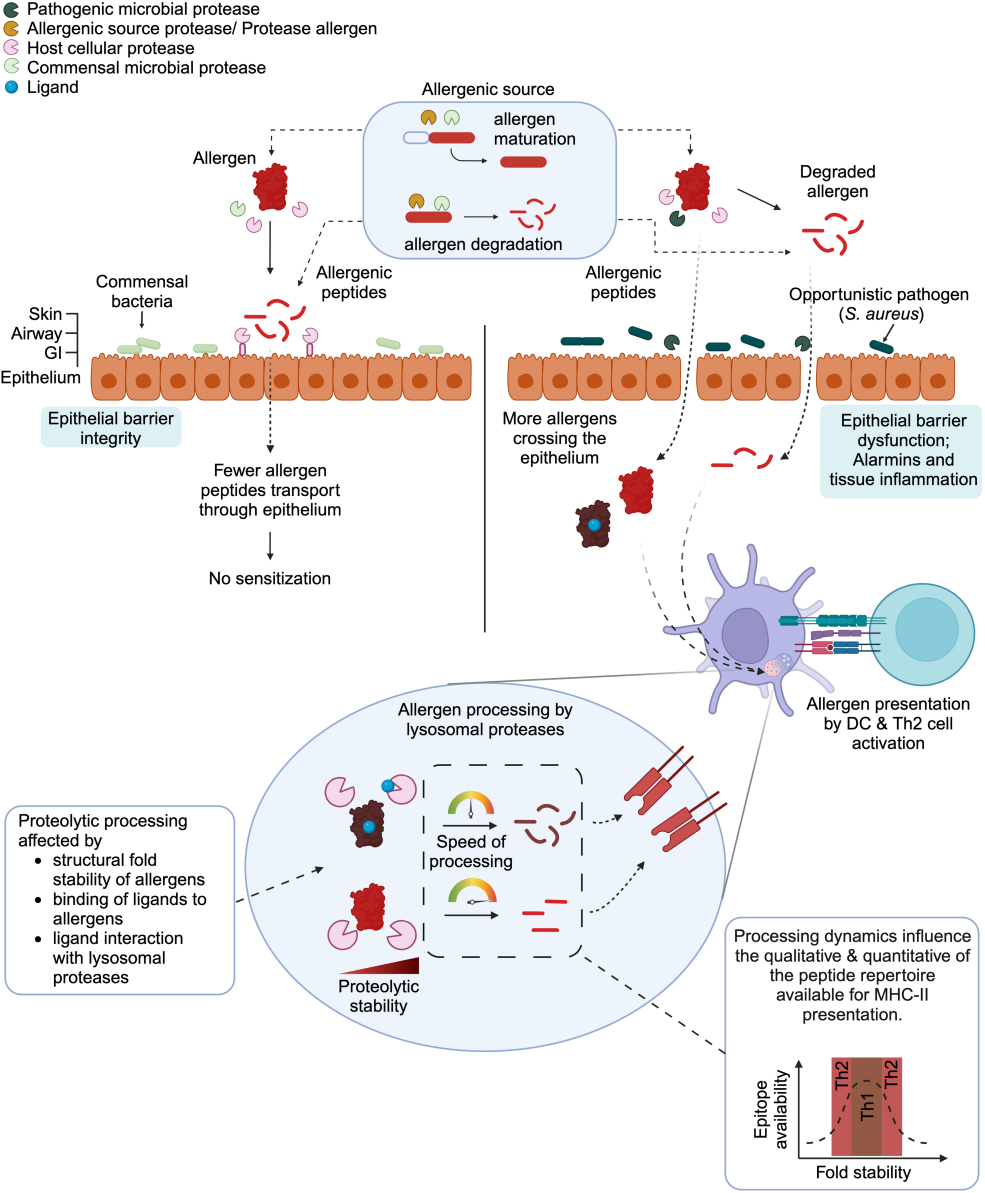
Effects of proteases on the fate of allergens. In their respective allergenic source (e.g., pollen, mite feces, food), allergens, once produced, can remain intact, are degraded or matured. Such events are mediated by non-allergenic proteases, protease allergens, or microbial proteases present in the allergenic sources. Upon entering into the sensitization route, intact allergens released from the allergenic sources can be cleaved by environmental (commensal and pathogenic microbes) or host cell proteases. (Left panel). Interactions between healthy epithelial barrier and commensal bacteria together with a fine-tuned protease/anti-protease system maintain epithelial integrity. Therefore, the allergens can be disarmed and fewer allergens/allergenic peptides can cross the epithelium resulting in the absence of sensitization. (Right panel) Conversely, epithelial barrier dysfunction, alarmins and tissue inflammation can be caused by opportunistic pathogens and result in the entry of allergens and allergenic peptides. The allergens or allergen-derived proteases can be taken up by antigen-presenting cells (e.g., dendritic cells) and presented to T helper cells, leading to Th2 polarization thanks to a pro- Th2 cytokine environment. (Bottom panel) Prior to presentation of allergenic T-cell epitopes, the allergens must be processed by lysosomal proteases from antigen presenting cells. Several factors are known to influence the proteolytic processing of allergens by lysosomal proteases. These include the structural fold stability of allergens, the presence of ligands (e.g., small lipids, metabolites) that bind to allergens or interact with lysosomal proteases. Consequently, these parameters alter the proteolytic processing dynamics, which then affect the quality and quantity of the peptide repertoire available for MHC-II presentation to T-cells. The influence of protein fold stability and epitope availability has been reviewed in details by Scheiblhofer et al. (72).

The spatiotemporal proteolytic stability of an allergen involves a complex interplay between the allergen's fold stability and the proteases present in its environment, i.e., proteases in the allergenic source and host proteases (Figure 1). It is generally accepted that an allergen requires stability against proteases to survive the entire journey to sensitization. Additionally, some allergens require proteases to assist in their maturation leading to their functionality. It has been shown that allergens are generally more intrinsically stable than non-allergens. External factors such as the redox environment, the availability of lipid ligands, and the presence of microbial proteases can influence the allergen proteolytic stability, thereby affecting its allergenicity. Investigating the antigen processing mediated by antigen-presenting cells provides another interesting aspect of proteolytic stability. How efficient is the allergen processing by lysosomal proteases can lead to changes at the immunological synapse between the antigen presenting cell and the T cell. Therefore, knowledges on the proteolytic stability required to drive the desired T cell polarization (Th2 or Th1/Th17) could be used for the development of new allergy therapeutics.

Several important questions about the proteolytic stability of allergens remain unanswered and require further investigations. Inflamed skin with a damaged barrier integrity is a potential site for food allergen sensitization. Skin barrier dysfunction is also a cardinal feature in the development of atopic dermatitis. Healthy skin harbors commensal bacteria such as *Staphylococcus epidermidis* and Corynebacterium species. As skin permeability increases, opportunistic pathogens such as *Staphylococcus aureus* dominate the microbial community and can colonize the skin (72). The effect of skin proteases, or proteases released by skin commensal or opportunistic pathogens, on allergens remains completely unexplored to date. We could speculate that in healthy skin with an intact barrier, allergens could be degraded by proteases secreted by keratinocytes or commensal bacteria. When the disruption of the epithelial barrier integrity is amplified by *Staphylococcus aureus* proteases, the rapid transit of allergens through the skin impairs their degradation.

The intestinal microbiota is rich in lactic acid bacteria, which express extracellular and membrane-bound proteases to gain access to amino acids, essential components for their cellular metabolism. It is well established that milk allergens can be degraded into polypeptides and amino acids by lactic acid bacterial proteases during milk fermentation. This food processing technique considerably reduces the milk allergenicity (73). Surprisingly, the effects of lactic acid bacterial proteases in the gut on the food allergens remain unknown and deserve in-depth investigations.

The airways contain a large variety of secreted (trypsin, chymotrypsin, elastase) and membrane-bound proteases from the airway epithelium [type II transmembrane serine proteases-TTSPs such as TMPRSS2, human airway trypsin-like protease (HAT) or matriptase] (74, 75). The proteolytic degradation of airborne allergens by these airway proteases is, to our knowledge, fully ignored. We cannot exclude that an allergen maturation mechanism is mediated by membrane-bound proteases, as observed for the proteolytic activation of SARS-Cov2 spike protein by TMPRSS2, which represents a key step for the viral entry into cells (76).

Activated mast cells were shown to release large amounts of tryptase. *in vitro* assays using physiological tryptase/allergen molar ratio showed complete degradation of pollen allergens Phl p 1, Phl p 6, and Bet v 2 (77). Under the same experimental conditions, pollen allergens Phl p 5 and Bet v 1 were fragmented. These cleavages/degradations allow the termination of the allergen-induced degranulation (77). It remains to be determined whether tryptase can cleave other allergens from other sources. Moreover, as other proteases are released by immune cells (elastase, cathepsin G released by neutrophils, granzyme B by basophils, chymase by mast cells), there is an urgent need for investigating the sensitivity of allergens to these proteases.
